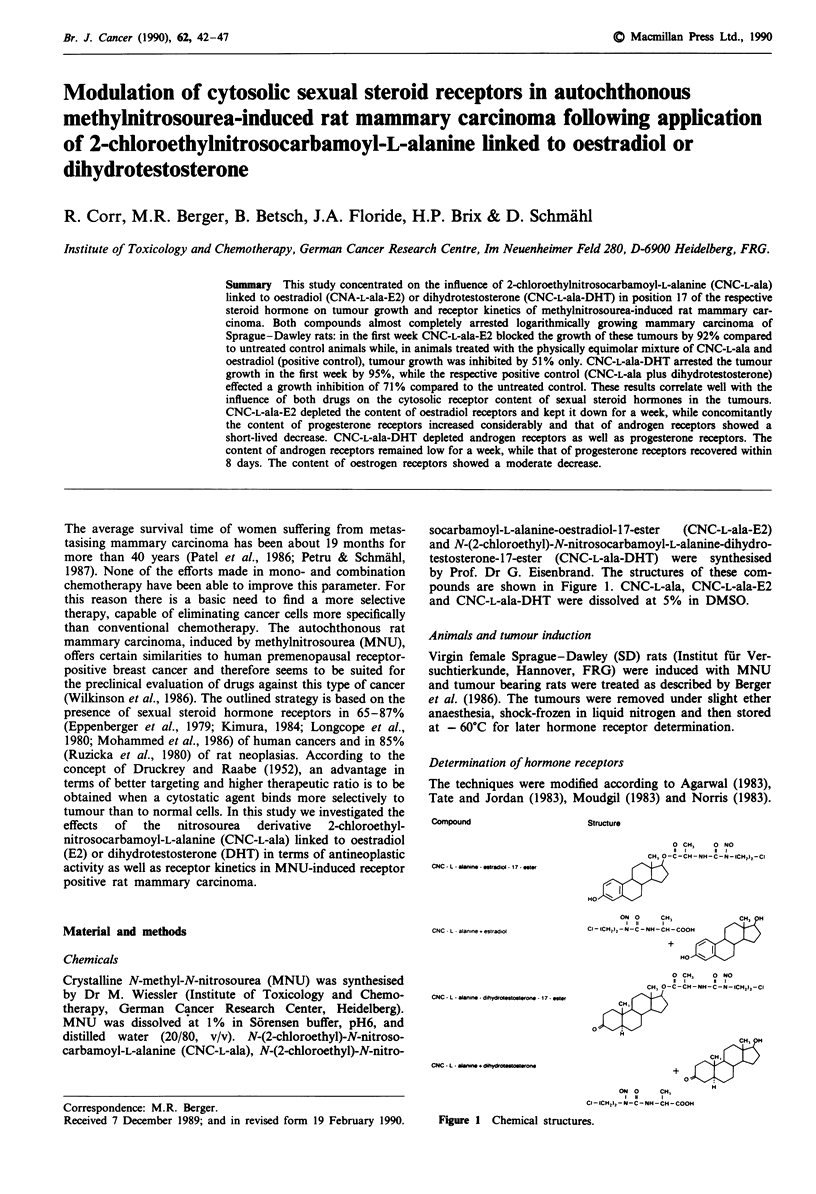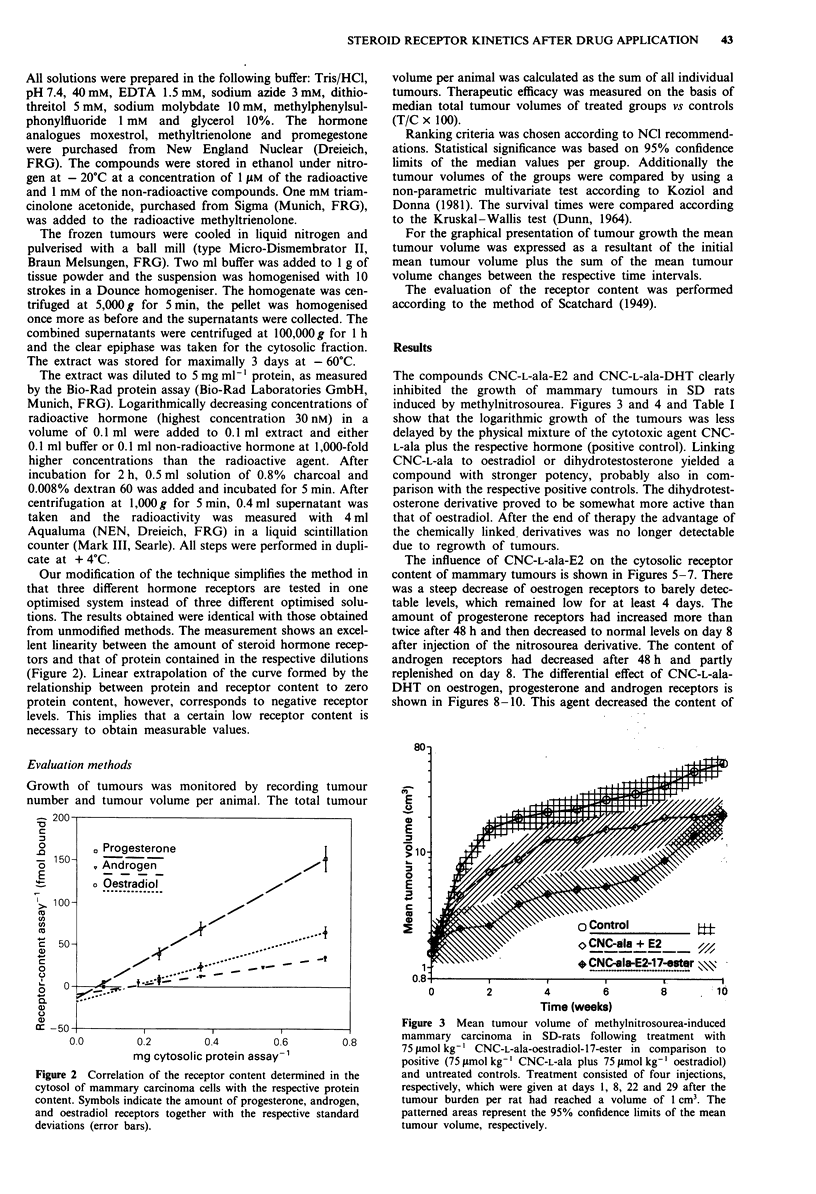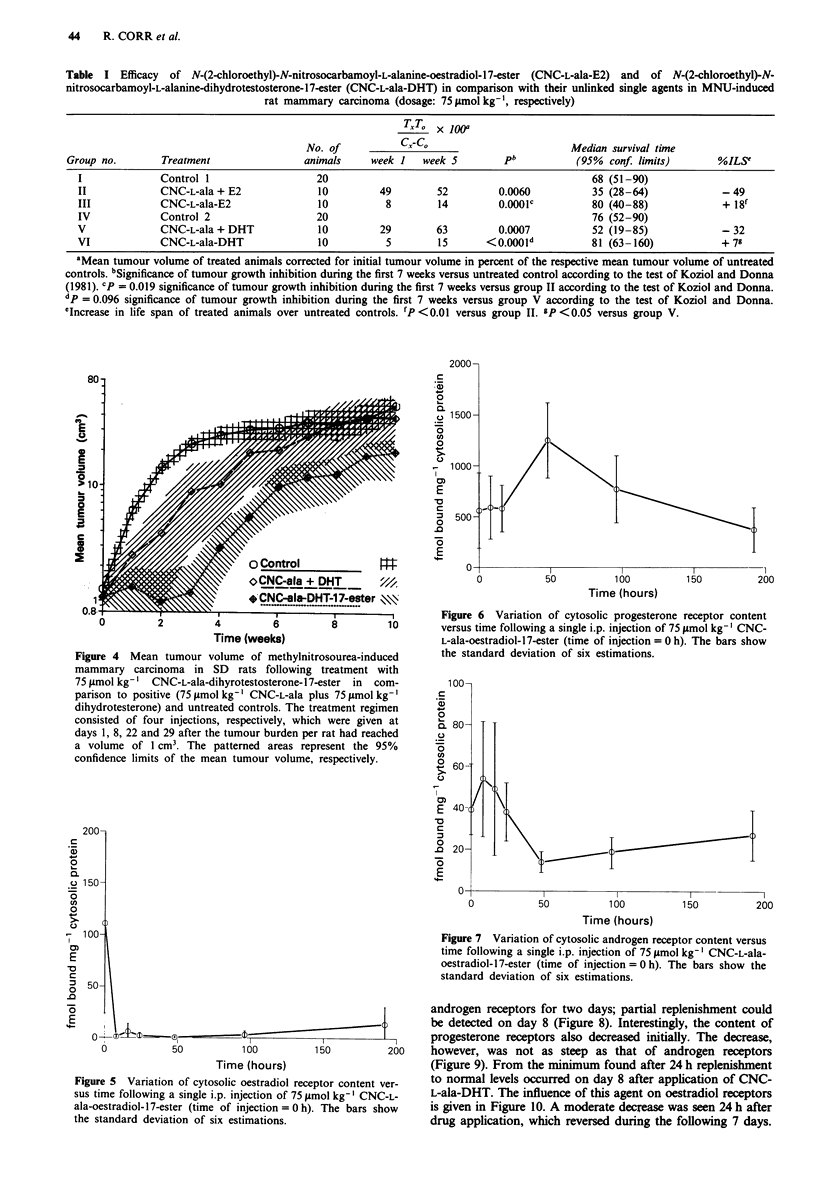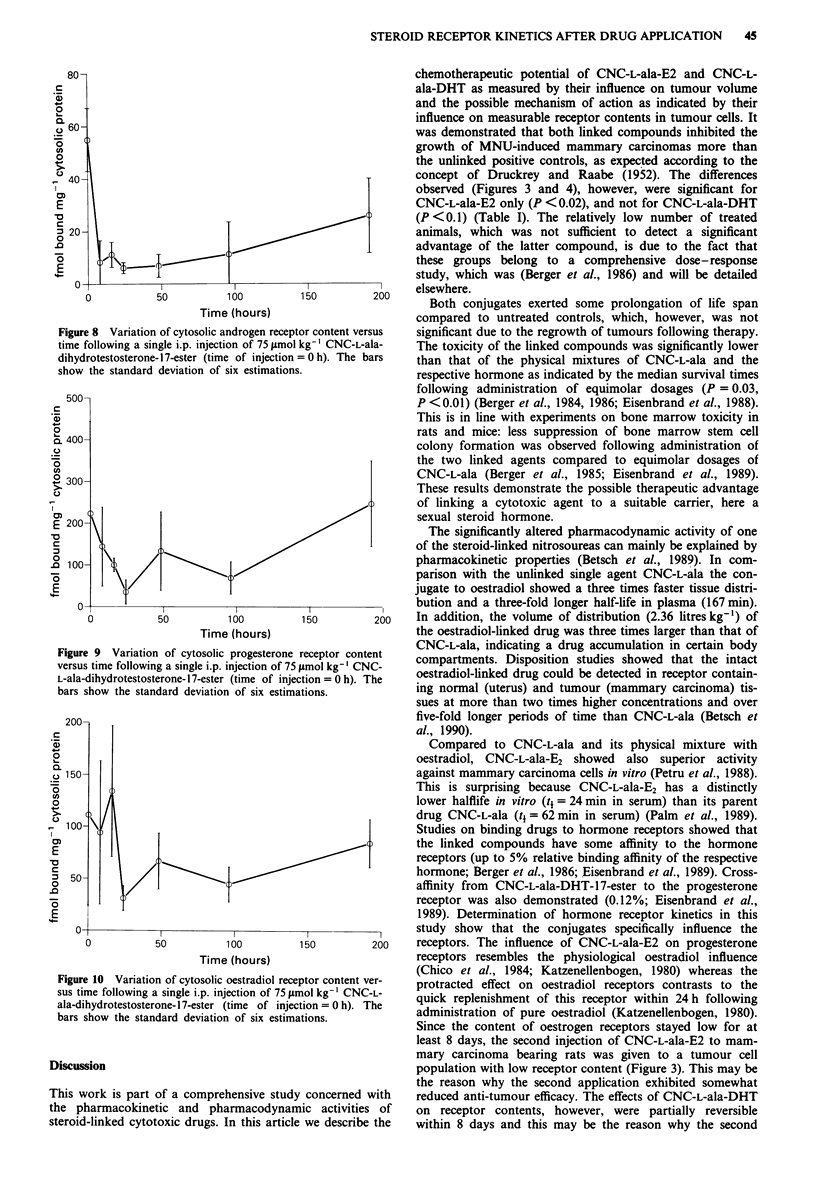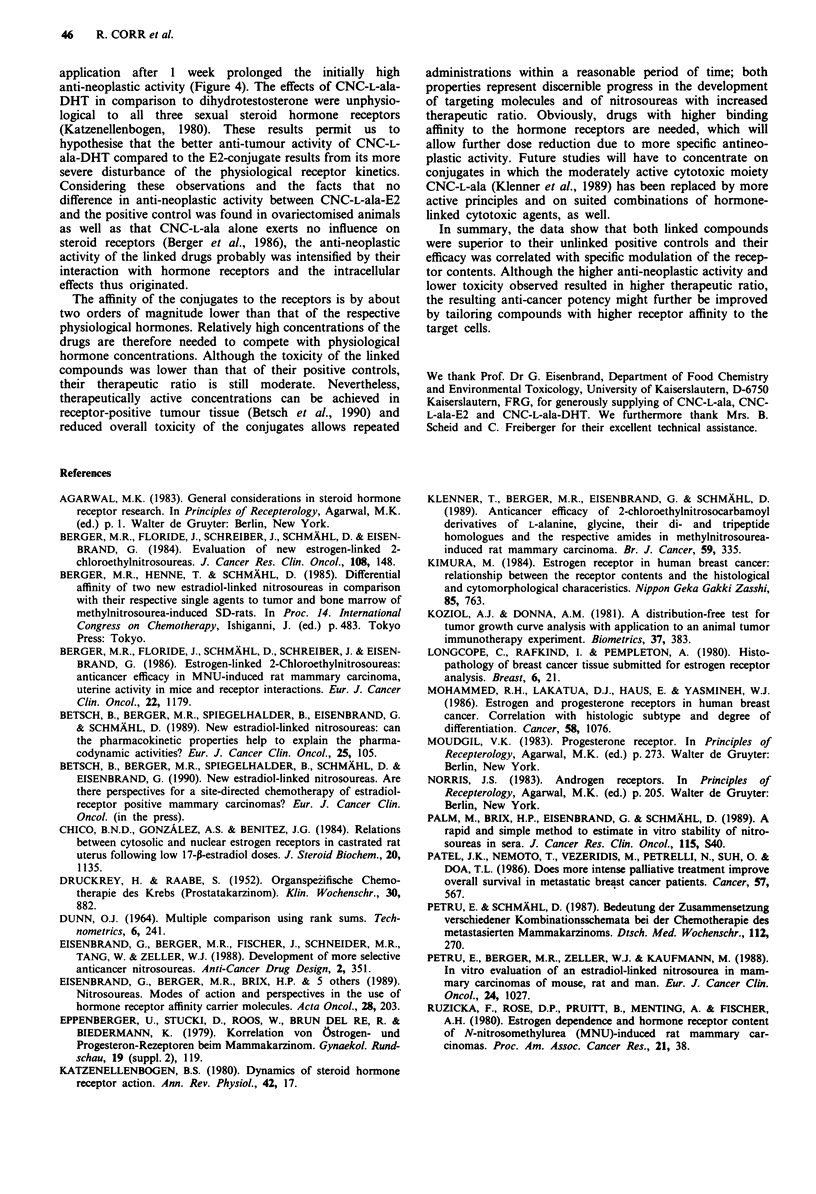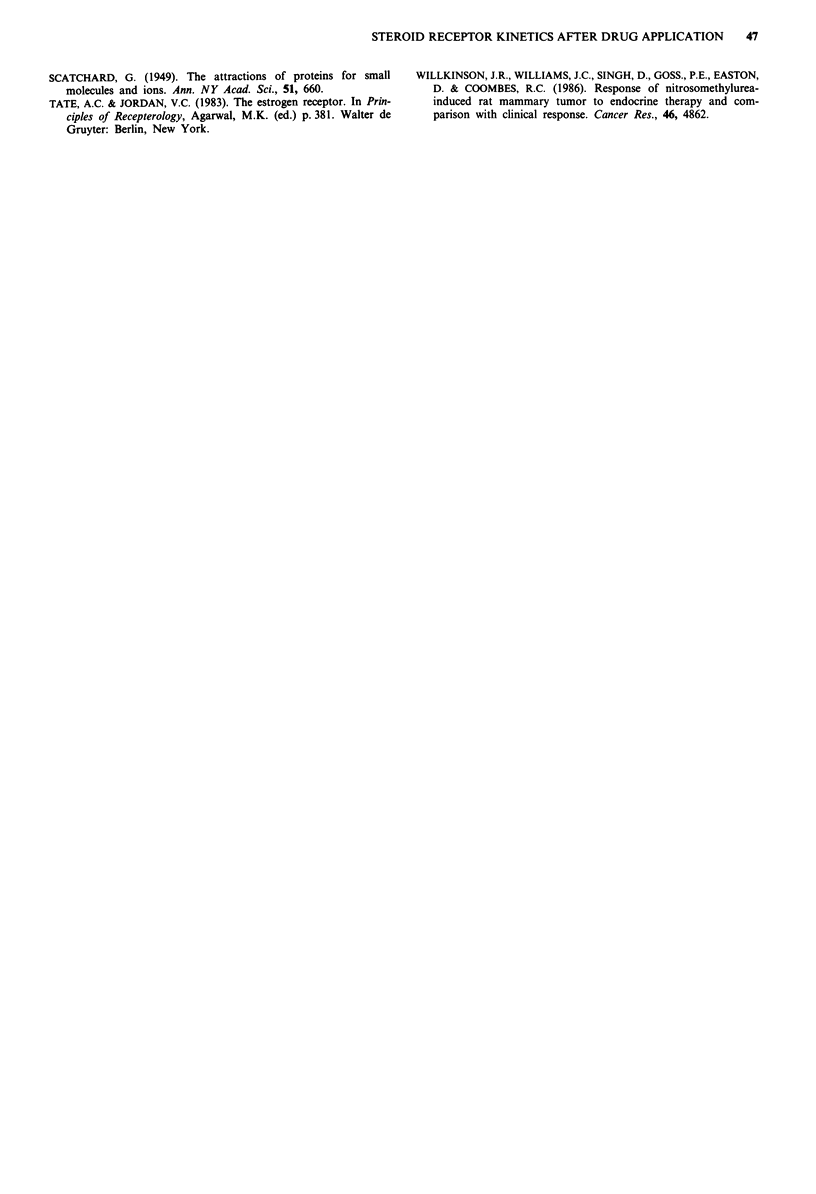# Modulation of cytosolic sexual steroid receptors in autochthonous methylnitrosourea-induced rat mammary carcinoma following application of 2-chloroethylnitrosocarbamoyl-L-alanine linked to oestradiol or dihydrotestosterone.

**DOI:** 10.1038/bjc.1990.226

**Published:** 1990-07

**Authors:** R. Corr, M. R. Berger, B. Betsch, J. A. Floride, H. P. Brix, D. Schmähl

**Affiliations:** Institute of Toxicology and Chemotherapy, German Cancer Research Centre, Heidelberg, FRG.

## Abstract

This study concentrated on the influence of 2-chloroethylnitrosocarbamoyl-L-alanine (CNC-L-ala) linked to oestradiol (CNA-L-ala-E2) or dihydrotestosterone (CNC-L-ala-DHT) in position 17 of the respective steroid hormone on tumour growth and receptor kinetics of methylnitrosourea-induced rat mammary carcinoma. Both compounds almost completely arrested logarithmically growing mammary carcinoma of Sprague-Dawley rats: in the first week CNC-L-ala-E2 blocked the growth of these tumours by 92% compared to untreated control animals while, in animals treated with the physically equimolar mixture of CNC-L-ala and oestradiol (positive control), tumour growth was inhibited by 51% only. CNC-L-ala-DHT arrested the tumour growth in the first week by 95%, while the respective positive control (CNC-L-ala plus dihydrotestosterone) effected a growth inhibition of 71% compared to the untreated control. These results correlate well with the influence of both drugs on the cytosolic receptor content of sexual steroid hormones in the tumours. CNC-L-ala-E2 depleted the content of oestradiol receptors and kept it down for a week, while concomitantly the content of progesterone receptors increased considerably and that of androgen receptors showed a short-lived decrease. CNC-L-ala-DHT depleted androgen receptors as well as progesterone receptors. The content of androgen receptors remained low for a week, while that of progesterone receptors recovered within 8 days. The content of oestrogen receptors showed a moderate decrease.


*Br. J. Cancer* (1990), **62**, 42–47

© Macmillan Press Ltd., 1990

### Modulation of cytosolic sexual steroid receptors in autochthonous methylnitrosourea-induced rat mammary carcinoma following application of 2-chloroethylnitrosocarbamoyl-L-alanine linked to oestradiol or dihydrotestosterone

R. Corr, M.R. Berger, B. Betsch, J.A. Floride, H.P. Brix & D. Schmähl

*Institute of Toxicology and Chemotherapy, German Cancer Research Centre, Im Neuenheimer Feld 280, D-6900 Heidelberg, FRG.*

**Summary** This study concentrated on the influence of 2-chloroethylnitrosocarbamoyl-L-alanine (CNC-L-ala) linked to oestradiol (CNA-L-ala-E2) or dihydrotestosterone (CNC-L-ala-DHT) in position 17 of the respective steroid hormone on tumour growth and receptor kinetics of methylnitrosourea-induced rat mammary carcinoma. Both compounds almost completely arrested logarithmically growing mammary carcinoma of Sprague–Dawley rats: in the first week CNC-L-ala-E2 blocked the growth of these tumours by 92% compared to untreated control animals while, in animals treated with the physically equimolar mixture of CNC-L-ala and oestradiol (positive control), tumour growth was inhibited by 51% only. CNC-L-ala-DHT arrested the tumour growth in the first week by 95%, while the respective positive control (CNC-L-ala plus dihydrotestosterone) effected a growth inhibition of 71% compared to the untreated control. These results correlate well with the influence of both drugs on the cytosolic receptor content of sexual steroid hormones in the tumours. CNC-L-ala-E2 depleted the content of oestradiol receptors and kept it down for a week, while concomitantly the content of progesterone receptors increased considerably and that of androgen receptors showed a short-lived decrease. CNC-L-ala-DHT depleted androgen receptors as well as progesterone receptors. The content of androgen receptors remained low for a week, while that of progesterone receptors recovered within 8 days. The content of oestrogen receptors showed a moderate decrease.

The average survival time of women suffering from metastasising mammary carcinoma has been about 19 months for more than 40 years (Patel *et al.*, 1986; Petru & Schmähl, 1987). None of the efforts made in mono- and combination chemotherapy have been able to improve this parameter. For this reason there is a basic need to find a more selective therapy, capable of eliminating cancer cells more specifically than conventional chemotherapy. The autochthonous rat mammary carcinoma, induced by methylnitrosourea (MNU), offers certain similarities to human premenopausal receptor-positive breast cancer and therefore seems to be suited for the preclinical evaluation of drugs against this type of cancer (Wilkinson *et al.*, 1986). The outlined strategy is based on the presence of sexual steroid hormone receptors in 65–87% (Eppenberger *et al.*, 1979; Kimura, 1984; Longcope *et al.*, 1980; Mohammed *et al.*, 1986) of human cancers and in 85% (Ruzicka *et al.*, 1980) of rat neoplasias. According to the concept of Druckrey and Raabe (1952), an advantage in terms of better targeting and higher therapeutic ratio is to be obtained when a cytostatic agent binds more selectively to tumour than to normal cells. In this study we investigated the effects of the nitrosourea derivative 2-chloroethyl-nitrosocarbamoyl-L-alanine (CNC-L-ala) linked to oestradiol (E2) or dihydrotestosterone (DHT) in terms of antineoplastic activity as well as receptor kinetics in MNU-induced receptor positive rat mammary carcinoma.

### Material and methods

### *Chemicals*

Crystalline *N*-methyl-*N*-nitrosourea (MNU) was synthesised by Dr M. Wiessler (Institute of Toxicology and Chemotherapy, German Cancer Research Center, Heidelberg). MNU was dissolved at 1% in Sörensen buffer, pH6, and distilled water (20/80, v/v). *N*-(2-chloroethyl)-*N*-nitrosocarbamoyl-L-alanine (CNC-L-ala), *N*-(2-chloroethyl)-*N*-nitrosocarbamoyl-L-alanine-oestradiol-17-ester (CNC-L-ala-E2) and *N*-(2-chloroethyl)-*N*-nitrosocarbamoyl-L-alanine-dihydrotestosterone-17-ester (CNC-L-ala-DHT) were synthesised by Prof. Dr G. Eisenbrand. The structures of these compounds are shown in Figure 1. CNC-L-ala, CNC-L-ala-E2 and CNC-L-ala-DHT were dissolved at 5% in DMSO.

### *Animals and tumour induction*

Virgin female Sprague–Dawley (SD) rats (Institut für Versuchtierkunde, Hannover, FRG) were induced with MNU and tumour bearing rats were treated as described by Berger *et al.* (1986). The tumours were removed under slight ether anaesthesia, shock-frozen in liquid nitrogen and then stored at − 60°C for later hormone receptor determination.

### *Determination of hormone receptors*

The techniques were modified according to Agarwal (1983), Tate and Jordan (1983), Moudgil (1983) and Norris (1983).

**Figure 1** Chemical structures.

Correspondence: M.R. Berger.
Received 7 December 1989; and in revised form 19 February 1990.

All solutions were prepared in the following buffer: Tris/HCl, pH 7.4, 40 mM, EDTA 1.5 mM, sodium azide 3 mM, dithiothreitol 5 mM, sodium molybdate 10 mM, methylphenylsulphonylfluoride 1 mM and glycerol 10%. The hormone analogues moxestrol, methyltrienolone and promegestone were purchased from New England Nuclear (Dreieich, FRG). The compounds were stored in ethanol under nitrogen at − 20°C at a concentration of 1 μM of the radioactive and 1 mM of the non-radioactive compounds. One mM triamcinolone acetonide, purchased from Sigma (Munich, FRG), was added to the radioactive methyltrienolone.

The frozen tumours were cooled in liquid nitrogen and pulverised with a ball mill (type Micro-Dismembrator II, Braun Melsungen, FRG). Two ml buffer was added to 1 g of tissue powder and the suspension was homogenised with 10 strokes in a Dounce homogeniser. The homogenate was centrifuged at 5,000 *g* for 5 min, the pellet was homogenised once more as before and the supernatants were collected. The combined supernatants were centrifuged at 100,000 *g* for 1 h and the clear epiphase was taken for the cytosolic fraction. The extract was stored for maximally 3 days at − 60°C.

The extract was diluted to 5 mg $ml^{-1}$ protein, as measured by the Bio-Rad protein assay (Bio-Rad Laboratories GmbH, Munich, FRG). Logarithmically decreasing concentrations of radioactive hormone (highest concentration 30 nM) in a volume of 0.1 ml were added to 0.1 ml extract and either 0.1 ml buffer or 0.1 ml non-radioactive hormone at 1,000-fold higher concentrations than the radioactive agent. After incubation for 2 h, 0.5 ml solution of 0.8% charcoal and 0.008% dextran 60 was added and incubated for 5 min. After centrifugation at 1,000 *g* for 5 min, 0.4 ml supernatant was taken and the radioactivity was measured with 4 ml Aqualuma (NEN, Dreieich, FRG) in a liquid scintillation counter (Mark III, Searle). All steps were performed in duplicate at + 4°C.

Our modification of the technique simplifies the method in that three different hormone receptors are tested in one optimised system instead of three different optimised solutions. The results obtained were identical with those obtained from unmodified methods. The measurement shows an excellent linearity between the amount of steroid hormone receptors and that of protein contained in the respective dilutions (Figure 2). Linear extrapolation of the curve formed by the relationship between protein and receptor content to zero protein content, however, corresponds to negative receptor levels. This implies that a certain low receptor content is necessary to obtain measurable values.

*Evaluation methods*

Growth of tumours was monitored by recording tumour number and tumour volume per animal. The total tumour

**Figure 2** Correlation of the receptor content determined in the cytosol of mammary carcinoma cells with the respective protein content. Symbols indicate the amount of progesterone, androgen, and oestradiol receptors together with the respective standard deviations (error bars).

volume per animal was calculated as the sum of all individual tumours. Therapeutic efficacy was measured on the basis of median total tumour volumes of treated groups *vs* controls (T/C × 100).

Ranking criteria was chosen according to NCl recommendations. Statistical significance was based on 95% confidence limits of the median values per group. Additionally the tumour volumes of the groups were compared by using a non-parametric multivariate test according to Koziol and Donna (1981). The survival times were compared according to the Kruskal–Wallis test (Dunn, 1964).

For the graphical presentation of tumour growth the mean tumour volume was expressed as a resultant of the initial mean tumour volume plus the sum of the mean tumour volume changes between the respective time intervals.

The evaluation of the receptor content was performed according to the method of Scatchard (1949).

### Results

The compounds CNC-L-ala-E2 and CNC-L-ala-DHT clearly inhibited the growth of mammary tumours in SD rats induced by methylnitrosourea. Figures 3 and 4 and Table I show that the logarithmic growth of the tumours was less delayed by the physical mixture of the cytotoxic agent CNC-L-ala plus the respective hormone (positive control). Linking CNC-L-ala to oestradiol or dihydrotestosterone yielded a compound with stronger potency, probably also in comparison with the respective positive controls. The dihydrotestosterone derivative proved to be somewhat more active than that of oestradiol. After the end of therapy the advantage of the chemically linked derivatives was no longer detectable due to regrowth of tumours.

The influence of CNC-L-ala-E2 on the cytosolic receptor content of mammary tumours is shown in Figures 5–7. There was a steep decrease of oestrogen receptors to barely detectable levels, which remained low for at least 4 days. The amount of progesterone receptors had increased more than twice after 48 h and then decreased to normal levels on day 8 after injection of the nitrosourea derivative. The content of androgen receptors had decreased after 48 h and partly replenished on day 8. The differential effect of CNC-L-ala-DHT on oestrogen, progesterone and androgen receptors is shown in Figures 8–10. This agent decreased the content of

**Figure 3** Mean tumour volume of methylnitrosourea-induced mammary carcinoma in SD-rats following treatment with 75 μmol $kg^{-1}$ CNC-L-ala-oestradiol-17-ester in comparison to positive (75 μmol $kg^{-1}$ CNC-L-ala plus 75 μmol $kg^{-1}$ oestradiol) and untreated controls. Treatment consisted of four injections, respectively, which were given at days 1, 8, 22 and 29 after the tumour burden per rat had reached a volume of 1 $cm^3$. The patterned areas represent the 95% confidence limits of the mean tumour volume, respectively.

**Table I** Efficacy of N-(2-chloroethyl)-N-nitrosocarbamoyl-L-alanine-oestradiol-17-ester (CNC-L-ala-E2) and of N-(2-chloroethyl)-N-nitrosocarbamoyl-L-alanine-dihydrotestosterone-17-ester (CNC-L-ala-DHT) in comparison with their unlinked single agents in MNU-induced rat mammary carcinoma (dosage: 75 μmol kg$^{-1}$, respectively)

| Group no. | Treatment | No. of animals | $\frac{T_xT_o}{C_x\text{-}C_o} \times 100^a$ week 1 | week 5 | $P^b$ | Median survival time (95% conf. limits) | %ILS[e] |
|---|---|---|---|---|---|---|---|
| I | Control 1 | 20 | | | | 68 (51-90) | |
| II | CNC-L-ala + E2 | 10 | 49 | 52 | 0.0060 | 35 (28-64) | -49 |
| III | CNC-L-ala-E2 | 10 | 8 | 14 | 0.0001[c] | 80 (40-88) | +18[f] |
| IV | Control 2 | 20 | | | | 76 (52-90) | |
| V | CNC-L-ala + DHT | 10 | 29 | 63 | 0.0007 | 52 (19-85) | -32 |
| VI | CNC-L-ala-DHT | 10 | 5 | 15 | <0.0001[d] | 81 (63-160) | +7[g] |

[a]Mean tumour volume of treated animals corrected for initial tumour volume in percent of the respective mean tumour volume of untreated controls. [b]Significance of tumour growth inhibition during the first 7 weeks versus untreated control according to the test of Koziol and Donna (1981). [c]$P = 0.019$ significance of tumour growth inhibition during the first 7 weeks versus group II according to the test of Koziol and Donna. [d]$P = 0.096$ significance of tumour growth inhibition during the first 7 weeks versus group V according to the test of Koziol and Donna. [e]Increase in life span of treated animals over untreated controls. [f]$P < 0.01$ versus group II. [g]$P < 0.05$ versus group V.

**Figure 4** Mean tumour volume of methylnitrosourea-induced mammary carcinoma in SD rats following treatment with 75 μmol kg$^{-1}$ CNC-L-ala-dihyrotestosterone-17-ester in comparison to positive (75 μmol kg$^{-1}$ CNC-L-ala plus 75 μmol kg$^{-1}$ dihydrotesterone) and untreated controls. The treatment regimen consisted of four injections, respectively, which were given at days 1, 8, 22 and 29 after the tumour burden per rat had reached a volume of 1 cm$^3$. The patterned areas represent the 95% confidence limits of the mean tumour volume, respectively.

**Figure 5** Variation of cytosolic oestradiol receptor content versus time following a single i.p. injection of 75 μmol kg$^{-1}$ CNC-L-ala-oestradiol-17-ester (time of injection = 0 h). The bars show the standard deviation of six estimations.

**Figure 6** Variation of cytosolic progesterone receptor content versus time following a single i.p. injection of 75 μmol kg$^{-1}$ CNC-L-ala-oestradiol-17-ester (time of injection = 0 h). The bars show the standard deviation of six estimations.

**Figure 7** Variation of cytosolic androgen receptor content versus time following a single i.p. injection of 75 μmol kg$^{-1}$ CNC-L-ala-oestradiol-17-ester (time of injection = 0 h). The bars show the standard deviation of six estimations.

androgen receptors for two days; partial replenishment could be detected on day 8 (Figure 8). Interestingly, the content of progesterone receptors also decreased initially. The decrease, however, was not as steep as that of androgen receptors (Figure 9). From the minimum found after 24 h replenishment to normal levels occurred on day 8 after application of CNC-L-ala-DHT. The influence of this agent on oestradiol receptors is given in Figure 10. A moderate decrease was seen 24 h after drug application, which reversed during the following 7 days.

**Figure 8** Variation of cytosolic androgen receptor content versus time following a single i.p. injection of 75 $\mu$mol $kg^{-1}$ CNC-L-ala-dihydrotestosterone-17-ester (time of injection = 0 h). The bars show the standard deviation of six estimations.

**Figure 9** Variation of cytosolic progesterone receptor content versus time following a single i.p. injection of 75 $\mu$mol $kg^{-1}$ CNC-L-ala-dihydrotestosterone-17-ester (time of injection = 0 h). The bars show the standard deviation of six estimations.

**Figure 10** Variation of cytosolic oestradiol receptor content versus time following a single i.p. injection of 75 $\mu$mol $kg^{-1}$ CNC-L-ala-dihydrotestosterone-17-ester (time of injection = 0 h). The bars show the standard deviation of six estimations.

### Discussion

This work is part of a comprehensive study concerned with the pharmacokinetic and pharmacodynamic activities of steroid-linked cytotoxic drugs. In this article we describe the chemotherapeutic potential of CNC-L-ala-E2 and CNC-L-ala-DHT as measured by their influence on tumour volume and the possible mechanism of action as indicated by their influence on measurable receptor contents in tumour cells. It was demonstrated that both linked compounds inhibited the growth of MNU-induced mammary carcinomas more than the unlinked positive controls, as expected according to the concept of Druckrey and Raabe (1952). The differences observed (Figures 3 and 4), however, were significant for CNC-L-ala-E2 only ($P < 0.02$), and not for CNC-L-ala-DHT ($P < 0.1$) (Table I). The relatively low number of treated animals, which was not sufficient to detect a significant advantage of the latter compound, is due to the fact that these groups belong to a comprehensive dose–response study, which was (Berger *et al.*, 1986) and will be detailed elsewhere.

Both conjugates exerted some prolongation of life span compared to untreated controls, which, however, was not significant due to the regrowth of tumours following therapy. The toxicity of the linked compounds was significantly lower than that of the physical mixtures of CNC-L-ala and the respective hormone as indicated by the median survival times following administration of equimolar dosages ($P = 0.03$, $P < 0.01$) (Berger *et al.*, 1984, 1986; Eisenbrand *et al.*, 1988). This is in line with experiments on bone marrow toxicity in rats and mice: less suppression of bone marrow stem cell colony formation was observed following administration of the two linked agents compared to equimolar dosages of CNC-L-ala (Berger *et al.*, 1985; Eisenbrand *et al.*, 1989). These results demonstrate the possible therapeutic advantage of linking a cytotoxic agent to a suitable carrier, here a sexual steroid hormone.

The significantly altered pharmacodynamic activity of one of the steroid-linked nitrosoureas can mainly be explained by pharmacokinetic properties (Betsch *et al.*, 1989). In comparison with the unlinked single agent CNC-L-ala the conjugate to oestradiol showed a three times faster tissue distribution and a three-fold longer half-life in plasma (167 min). In addition, the volume of distribution (2.36 litres $kg^{-1}$) of the oestradiol-linked drug was three times larger than that of CNC-L-ala, indicating a drug accumulation in certain body compartments. Disposition studies showed that the intact oestradiol-linked drug could be detected in receptor containing normal (uterus) and tumour (mammary carcinoma) tissues at more than two times higher concentrations and over five-fold longer periods of time than CNC-L-ala (Betsch *et al.*, 1990).

Compared to CNC-L-ala and its physical mixture with oestradiol, CNC-L-ala-$E_2$ showed also superior activity against mammary carcinoma cells *in vitro* (Petru *et al.*, 1988). This is surprising because CNC-L-ala-$E_2$ has a distinctly lower halflife *in vitro* ($t_{1/2}$ = 24 min in serum) than its parent drug CNC-L-ala ($t_{1/2}$ = 62 min in serum) (Palm *et al.*, 1989). Studies on binding drugs to hormone receptors showed that the linked compounds have some affinity to the hormone receptors (up to 5% relative binding affinity of the respective hormone; Berger *et al.*, 1986; Eisenbrand *et al.*, 1989). Cross-affinity from CNC-L-ala-DHT-17-ester to the progesterone receptor was also demonstrated (0.12%; Eisenbrand *et al.*, 1989). Determination of hormone receptor kinetics in this study show that the conjugates specifically influence the receptors. The influence of CNC-L-ala-E2 on progesterone receptors resembles the physiological oestradiol influence (Chico *et al.*, 1984; Katzenellenbogen, 1980) whereas the protracted effect on oestradiol receptors contrasts to the quick replenishment of this receptor within 24 h following administration of pure oestradiol (Katzenellenbogen, 1980). Since the content of oestrogen receptors stayed low for at least 8 days, the second injection of CNC-L-ala-E2 to mammary carcinoma bearing rats was given to a tumour cell population with low receptor content (Figure 3). This may be the reason why the second application exhibited somewhat reduced anti-tumour efficacy. The effects of CNC-L-ala-DHT on receptor contents, however, were partially reversible within 8 days and this may be the reason why the second application after 1 week prolonged the initially high anti-neoplastic activity (Figure 4). The effects of CNC-L-ala-DHT in comparison to dihydrotestosterone were unphysiological to all three sexual steroid hormone receptors (Katzenellenbogen, 1980). These results permit us to hypothesise that the better anti-tumour activity of CNC-L-ala-DHT compared to the E2-conjugate results from its more severe disturbance of the physiological receptor kinetics. Considering these observations and the facts that no difference in anti-neoplastic activity between CNC-L-ala-E2 and the positive control was found in ovariectomised animals as well as that CNC-L-ala alone exerts no influence on steroid receptors (Berger *et al.*, 1986), the anti-neoplastic activity of the linked drugs probably was intensified by their interaction with hormone receptors and the intracellular effects thus originated.

The affinity of the conjugates to the receptors is by about two orders of magnitude lower than that of the respective physiological hormones. Relatively high concentrations of the drugs are therefore needed to compete with physiological hormone concentrations. Although the toxicity of the linked compounds was lower than that of their positive controls, their therapeutic ratio is still moderate. Nevertheless, therapeutically active concentrations can be achieved in receptor-positive tumour tissue (Betsch *et al.*, 1990) and reduced overall toxicity of the conjugates allows repeated administrations within a reasonable period of time; both properties represent discernible progress in the development of targeting molecules and of nitrosoureas with increased therapeutic ratio. Obviously, drugs with higher binding affinity to the hormone receptors are needed, which will allow further dose reduction due to more specific antineoplastic activity. Future studies will have to concentrate on conjugates in which the moderately active cytotoxic moiety CNC-L-ala (Klenner *et al.*, 1989) has been replaced by more active principles and on suited combinations of hormone-linked cytotoxic agents, as well.

In summary, the data show that both linked compounds were superior to their unlinked positive controls and their efficacy was correlated with specific modulation of the receptor contents. Although the higher anti-neoplastic activity and lower toxicity observed resulted in higher therapeutic ratio, the resulting anti-cancer potency might further be improved by tailoring compounds with higher receptor affinity to the target cells.

We thank Prof. Dr G. Eisenbrand, Department of Food Chemistry and Environmental Toxicology, University of Kaiserslautern, D-6750 Kaiserslautern, FRG, for generously supplying of CNC-L-ala, CNC-L-ala-E2 and CNC-L-ala-DHT. We furthermore thank Mrs. B. Scheid and C. Freiberger for their excellent technical assistance.

### References

AGARWAL, M.K. (1983). General considerations in steroid hormone receptor research. In *Principles of Recepterology*, Agarwal, M.K. (ed.) p. 1. Walter de Gruyter: Berlin, New York.

BERGER, M.R., FLORIDE, J., SCHREIBER, J., SCHMÄHL, D. & EISENBRAND, G. (1984). Evaluation of new estrogen-linked 2-chloroethylnitrosoureas. *J. Cancer Res. Clin. Oncol.*, **108**, 148.

BERGER, M.R., HENNE, T. & SCHMÄHL, D. (1985). Differential affinity of two new estradiol-linked nitrosoureas in comparison with their respective single agents to tumor and bone marrow of methylnitrosourea-induced SD-rats. In *Proc. 14. International Congress on Chemotherapy*, Ishiganni, J. (ed.) p. 483. Tokyo Press: Tokyo.

BERGER, M.R., FLORIDE, J., SCHMÄHL, D., SCHREIBER, J. & EISENBRAND, G. (1986). Estrogen-linked 2-Chloroethylnitrosoureas: anticancer efficacy in MNU-induced rat mammary carcinoma, uterine activity in mice and receptor interactions. *Eur. J. Cancer Clin. Oncol.*, **22**, 1179.

BETSCH, B., BERGER, M.R., SPIEGELHALDER, B., EISENBRAND, G. & SCHMÄHL, D. (1989). New estradiol-linked nitrosoureas: can the pharmacokinetic properties help to explain the pharmacodynamic activities? *Eur. J. Cancer Clin. Oncol.*, **25**, 105.

BETSCH, B., BERGER, M.R., SPIEGELHALDER, B., SCHMÄHL, D. & EISENBRAND, G. (1990). New estradiol-linked nitrosoureas. Are there perspectives for a site-directed chemotherapy of estradiol-receptor positive mammary carcinomas? *Eur. J. Cancer Clin. Oncol.* (in the press).

CHICO, B.N.D., GONZÁLEZ, A.S. & BENITEZ, J.G. (1984). Relations between cytosolic and nuclear estrogen receptors in castrated rat uterus following low 17-β-estradiol doses. *J. Steroid Biochem.*, **20**, 1135.

DRUCKREY, H. & RAABE, S. (1952). Organspezifische Chemotherapie des Krebs (Prostatakarzinom). *Klin. Wochenschr.*, **30**, 882.

DUNN, O.J. (1964). Multiple comparison using rank sums. *Technometrics*, **6**, 241.

EISENBRAND, G., BERGER, M.R., FISCHER, J., SCHNEIDER, M.R., TANG, W. & ZELLER, W.J. (1988). Development of more selective anticancer nitrosoureas. *Anti-Cancer Drug Design*, **2**, 351.

EISENBRAND, G., BERGER, M.R., BRIX, H.P. & 5 others (1989). Nitrosoureas. Modes of action and perspectives in the use of hormone receptor affinity carrier molecules. *Acta Oncol.*, **28**, 203.

EPPENBERGER, U., STUCKI, D., ROOS, W., BRUN DEL RE, R. & BIEDERMANN, K. (1979). Korrelation von Östrogen- und Progesteron-Rezeptoren beim Mammakarzinom. *Gynaekol. Rundschau*, **19** (suppl. 2), 119.

KATZENELLENBOGEN, B.S. (1980). Dynamics of steroid hormone receptor action. *Ann. Rev. Physiol.*, **42**, 17.

KLENNER, T., BERGER, M.R., EISENBRAND, G. & SCHMÄHL, D. (1989). Anticancer efficacy of 2-chloroethylnitrosocarbamoyl derivatives of L-alanine, glycine, their di- and tripeptide homologues and the respective amides in methylnitrosourea-induced rat mammary carcinoma. *Br. J. Cancer*, **59**, 335.

KIMURA, M. (1984). Estrogen receptor in human breast cancer: relationship between the receptor contents and the histological and cytomorphological characeristics. *Nippon Geka Gakki Zasshi*, **85**, 763.

KOZIOL, A.J. & DONNA, A.M. (1981). A distribution-free test for tumor growth curve analysis with application to an animal tumor immunotherapy experiment. *Biometrics*, **37**, 383.

LONGCOPE, C., RAFKIND, I. & PEMPLETON, A. (1980). Histopathology of breast cancer tissue submitted for estrogen receptor analysis. *Breast*, **6**, 21.

MOHAMMED, R.H., LAKATUA, D.J., HAUS, E. & YASMINEH, W.J. (1986). Estrogen and progesterone receptors in human breast cancer. Correlation with histologic subtype and degree of differentiation. *Cancer*, **58**, 1076.

MOUDGIL, V.K. (1983). Progesterone receptor. In *Principles of Recepterology*, Agarwal, M.K. (ed.) p. 273. Walter de Gruyter: Berlin, New York.

NORRIS, J.S. (1983). Androgen receptors. In *Principles of Recepterology*, Agarwal, M.K. (ed.) p. 205. Walter de Gruyter: Berlin, New York.

PALM, M., BRIX, H.P., EISENBRAND, G. & SCHMÄHL, D. (1989). A rapid and simple method to estimate in vitro stability of nitrosoureas in sera. *J. Cancer Res. Clin. Oncol.*, **115**, S40.

PATEL, J.K., NEMOTO, T., VEZERIDIS, M., PETRELLI, N., SUH, O. & DOA, T.L. (1986). Does more intense palliative treatment improve overall survival in metastatic breast cancer patients. *Cancer*, **57**, 567.

PETRU, E. & SCHMÄHL, D. (1987). Bedeutung der Zusammensetzung verschiedener Kombinationsschemata bei der Chemotherapie des metastasierten Mammakarzinoms. *Dtsch. Med. Wochenschr.*, **112**, 270.

PETRU, E., BERGER, M.R., ZELLER, W.J. & KAUFMANN, M. (1988). In vitro evaluation of an estradiol-linked nitrosourea in mammary carcinomas of mouse, rat and man. *Eur. J. Cancer Clin. Oncol.*, **24**, 1027.

RUZICKA, F., ROSE, D.P., PRUITT, B., MENTING, A. & FISCHER, A.H. (1980). Estrogen dependence and hormone receptor content of *N*-nitrosomethylurea (MNU)-induced rat mammary carcinomas. *Proc. Am. Assoc. Cancer Res.*, **21**, 38.

SCATCHARD, G. (1949). The attractions of proteins for small molecules and ions. *Ann. NY Acad. Sci.*, **51**, 660.

TATE, A.C. & JORDAN, V.C. (1983). The estrogen receptor. In *Principles of Recepterology*, Agarwal, M.K. (ed.) p. 381. Walter de Gruyter: Berlin, New York.

WILLKINSON, J.R., WILLIAMS, J.C., SINGH, D., GOSS., P.E., EASTON, D. & COOMBES, R.C. (1986). Response of nitrosomethylurea-induced rat mammary tumor to endocrine therapy and comparison with clinical response. *Cancer Res.*, **46**, 4862.